# Potential risks of Oropouche virus importation into Europe

**DOI:** 10.1093/jtm/taae109

**Published:** 2024-08-19

**Authors:** Maria R Capobianchi, Concetta Castilletti, Federico Giovanni Gobbi

**Affiliations:** Department of Infectious-Tropical Diseases and Microbiology, IRCCS Sacro Cuore Don Calabria Hospital, Negrar di Valpolicella, Verona, Italy; Department of Infectious-Tropical Diseases and Microbiology, IRCCS Sacro Cuore Don Calabria Hospital, Negrar di Valpolicella, Verona, Italy; Department of Infectious-Tropical Diseases and Microbiology, IRCCS Sacro Cuore Don Calabria Hospital, Negrar di Valpolicella, Verona, Italy; Department of Clinical and Experimental Sciences, University of Brescia, Brescia, Italy

## Abstract

Imported cases of Oropouche fever were recently detected in Italy. Upcoming mass events, i.e. the 2024 Olympic Games in Paris and the 2025 Jubilee in Rome, represent increasing likelihood of further OROV importation and potential spread in new areas, underscoring the importance of strengthening surveillance, laboratory capacity and ecology studies.

Increased long distance movements of people increase the risk of spreading pathogens originating from the tropics to the so-called temperate climatic zones. On the other hand, progressive global warming fosters the expansion of potential arthropod vectors to previously spared regions. As a result, a number of viruses previously confined to tropical regions are expanding their geographic range and are establishing autochthonous cycles in new areas.^1–4^ This is indeed the case of West Nile virus (WNV), and the much less pathogenic USUTU virus (USUV). Birds are the primary amplifying hosts for both viruses, and transmission cycles involve ornithophilic mosquitoes and resident or migratory birds. Both viruses have become endemic in several European countries, particularly in Italy, where the main mosquito vector for humans, *Culex pipiens*, is widely distributed,^5^^,^^6^ and amplifying hosts are present.

Increasing frequency of other arboviruses importation, well adapted to vector-mediated human-to-human transmission, such as dengue (DENV) and Chikungunya viruses (CHIKV), lead to the occurrence of small to medium scale outbreaks in European regions where the local vector, *Aedes albopictus*, is widely diffused.^7^^,^^8^

A new emerging arbovirus, Oropouche virus (OROV), for which there is still little awareness, is reaching the spotlight in these days. Culicoides are considered the preferred vectors of this virus for humans and animals. In particular, *Culicoides paraensis* is considered ‘a neglected vector for a neglected disease’; mosquitoes such as *Culex quinquefasciatus* and *Aedes serratus*—and perhaps other arthropods—can also be competent vectors.^9^ Overall, vector competence for OROV is still largely unexplored. Yet, a relevant aspect to address is to establish whether OROV involved in the present outbreak has developed the ability to overcome the midgut barrier in widely diffused putative vectors, that is a pre-requisite to develop a disseminated infection and, in turn, to increase the vector(s) ability to transmit the infection to humans.^10^

The clinical spectrum of OROV infection manifestations may range from mild febrile illness to severe disease, including neurological and haemorrhagic outcomes. So far, no deaths attributed to OROV are reported, but underreporting of OROV infections is likely due to similarity with DENV infection symptoms; moreover, OROV diagnosis can be performed only in specialized laboratories.

A sustained outbreak of OROV is occurring in Central and South America, with ~5000 cases reported in Brazil. On 27 May 2024, the Ministry of Public Health of Cuba reported the first ever outbreak of OROV disease in this island.^11^

Until recently, no OROV infections outside of the Americas had been reported: however, considering the ongoing environmental and climate changes, and intensification of human travel on a global scale, OROV is likely to spread outside of South America in the near future. In fact, in late May–early June 2024, OROV infection has been reported in two epidemiologically unrelated travellers returning to Italy from Cuba. To our knowledge this is the first ever report of OROV infection outside of Latin America and should be regarded as an alarming bell for what we can expect for the epidemiology of OROV in the future.^12^

Large human movements and mass gathering events typically offer to emerging viruses the opportunity to spread and expand their geographic diffusion. To properly assess the risk of further importation of OROV cases from countries where an outbreak is occurring, it has to be considered that an average of 39 000 flight passengers arrived from Cuba to Europe every month during the first 4 months of 2024, based on data from the International Air Travel Association (IATA); in the last 2 years peak frequency was reported in August, with ~49 000 passengers arrived from Cuba.^13^ In addition to this routine traffic, in the near future some events will involve millions of people travelling to Europe: tourists and athletes from all around the world will attend the Olympic Games scheduled to take place in Paris in July/August 2024. Moreover, millions of pilgrims will travel to Rome, Italy, to attend the rituals of the Jubilee that will start on Christmas Eve 2024 with the opening of the Holy Door in St. Peter’s Basilica in the Vatican, and will end on Epiphany 2026. In the tradition of the Catholic Church, during the Jubilee or Holy Year pilgrims visiting the holy places of Christianity, especially in the city of Rome, obtain plenary indulgence. The first Jubilee was called in 1300 by Pope Boniface VIII, who established a 100-year cadence between Jubilees, later reduced to 50, 33 and finally 25 years.^14^

The 2015 previous Jubilee took to Rome >20 millions of pilgrims from 156 countries around the world, and for the 2025 event >30 millions of pilgrims are forecasted.^15^

Latin America countries, where Catholicism is the most widespread religion, are significant contributors to the visitor’s flow to Rome religious events. This means a high probability that multiple introductions of OROV to Italy from the affected territories could occur in the near future. Furthermore, the mass gathering events that will occur during this event will represent a mixing basin where people from multiple countries will stay together, increasing the opportunities for the virus to gain new geographic regions.

Overall, increasing the likelihood of OROV emergence in new areas underscores its importance on an international public health scale. A robust and ready to react surveillance system will be required to promptly intercept infected people and adopt protection measures to avoid that insect bites may ignite a vicious circle, leading to autochthonous transmission of OROV and establishment of infection clusters, and, eventually, geographic spread. In order to stem the spread of OROV, in addition to vector proficiency studies and strengthening laboratory capabilities, it will be crucial to raise awareness among healthcare professionals to place OROV in the diagnostic landscape of imported fevers.
